# Anxiety and depression in patients undergoing total laryngectomy

**DOI:** 10.1192/j.eurpsy.2025.1793

**Published:** 2025-08-26

**Authors:** N. Sghaier, W. Zaier, R. Ben Soussia, W. Bouali, F. Youzbechi, S. Ben Hadj Said, L. Zarrouk

**Affiliations:** 1 Psychiatry Departement, Ibn Jazzar Hospital, Kairouan; 2 Psychiatry Departement, Tahar Sfar Hospital, Mahdia, Tunisia

## Abstract

**Introduction:**

Total laryngectomy is a major surgical procedure involving the complete removal of the larynx, often necessary to treat severe conditions such as laryngeal cancer. Although this operation is often life-saving, it leads to significant and often disabling consequences that impact mental health, including increased levels of anxiety and depression.

**Objectives:**

Determine the prevalence of anxiety and depression among a group of patients who have undergone total laryngectomy and to investigate the factors associated with their occurrence.

**Methods:**

This is a descriptive cross-sectional study with an analytical aim conducted over a period of 6 months from December 2021 to June 2022, involving patients followed for laryngeal cancer who had undergone total laryngectomy at the Otolaryngology department of Hospital Tahar Sfar Mahdia. Anxiety and depression were assessed using the Hamilton Anxiety Rating Scale and the Beck Depression Inventory , respectively.

**Results:**

A total of 40 patients participated in the study. The average age was 62±9 years, with a population consisting of 100% males. A score greater than 20 indicating the presence of anxiety symptoms was noted in 50% of patients. Depression was noted in 78% of patients, with moderate intensity in 42% and severe in 25% of cases. We found that shorter postoperative follow-up was associated with greater anxiety symptoms (p=0.005). However, other factors such as diabetes and impact on social life could be related to anxiety, though no statistically significant associations were found. Regarding depressive symptoms, only the presence of postoperative complications was statistically related to moderate or severe depressive symptoms (p=0.004) in our study. Other factors such as radiotherapy and impact on work might be related to depressive symptoms, although no statistically significant associations were found.

**Conclusions:**

Total laryngectomy has undeniable repercussions on the mental health of patients. Medical and social support is essential to help them better cope with this disability.

**Disclosure of Interest:**

None Declared

